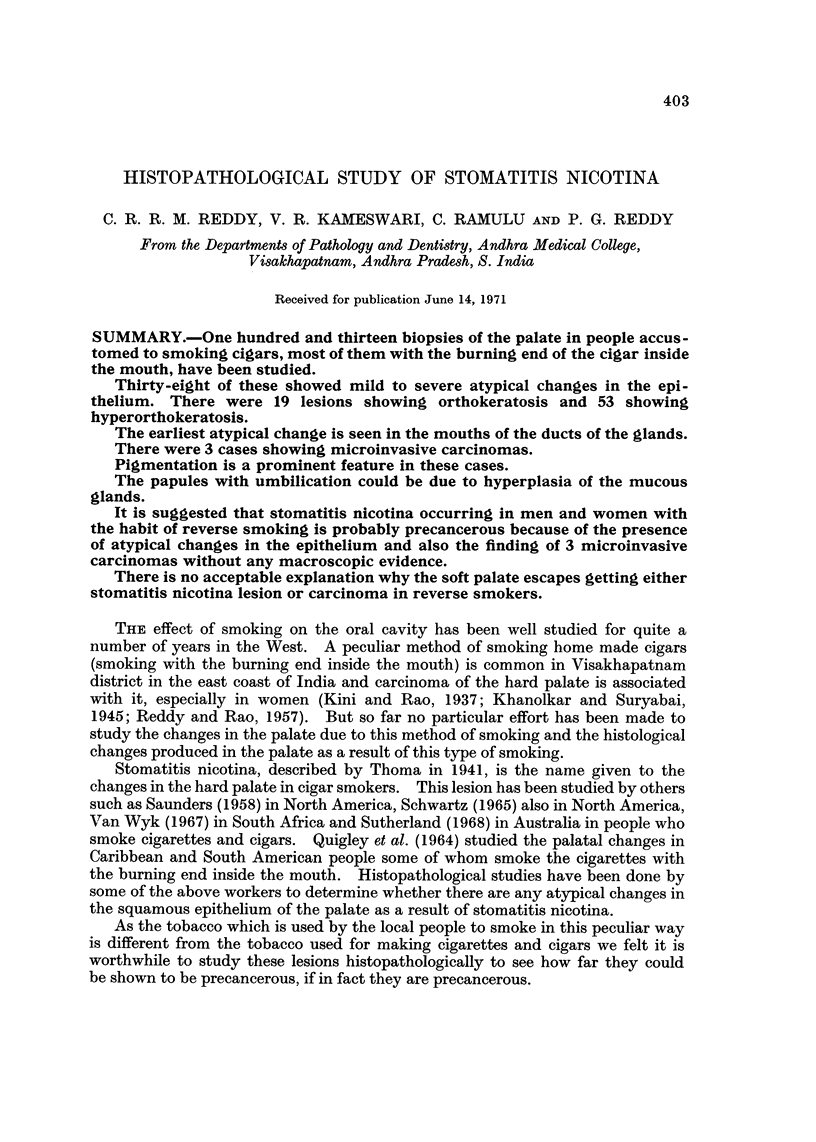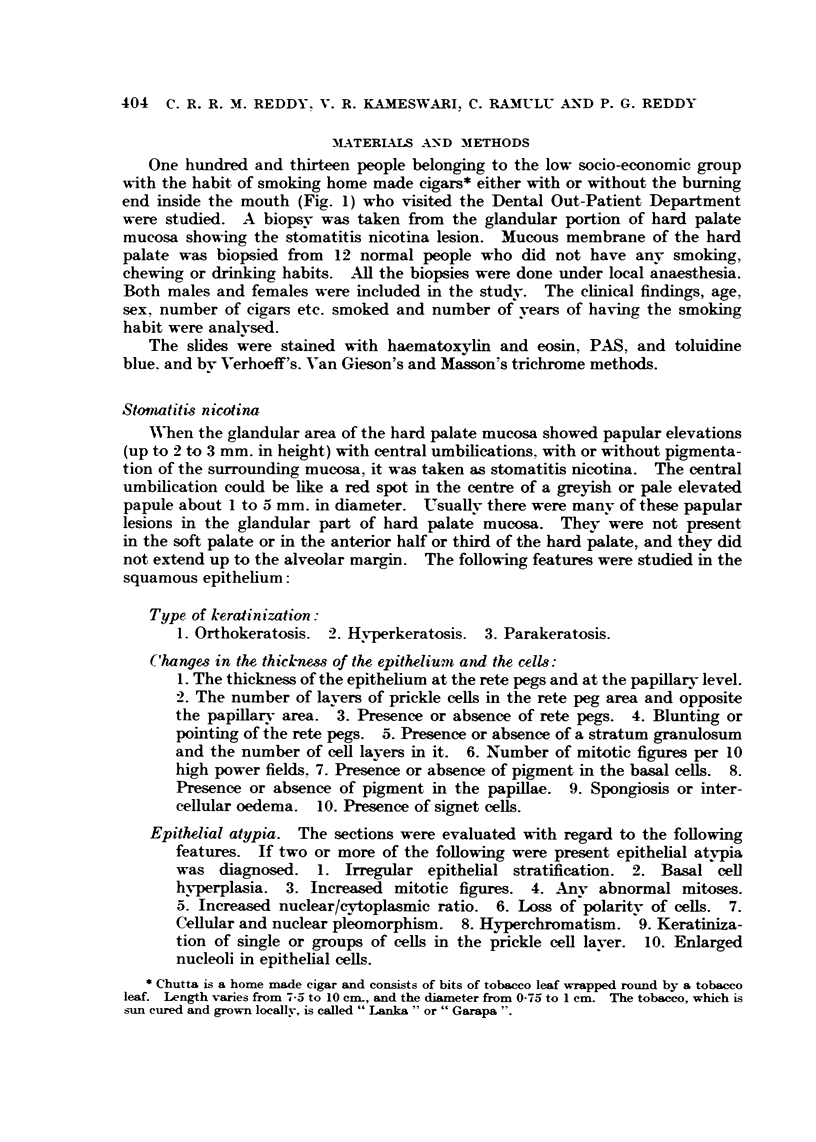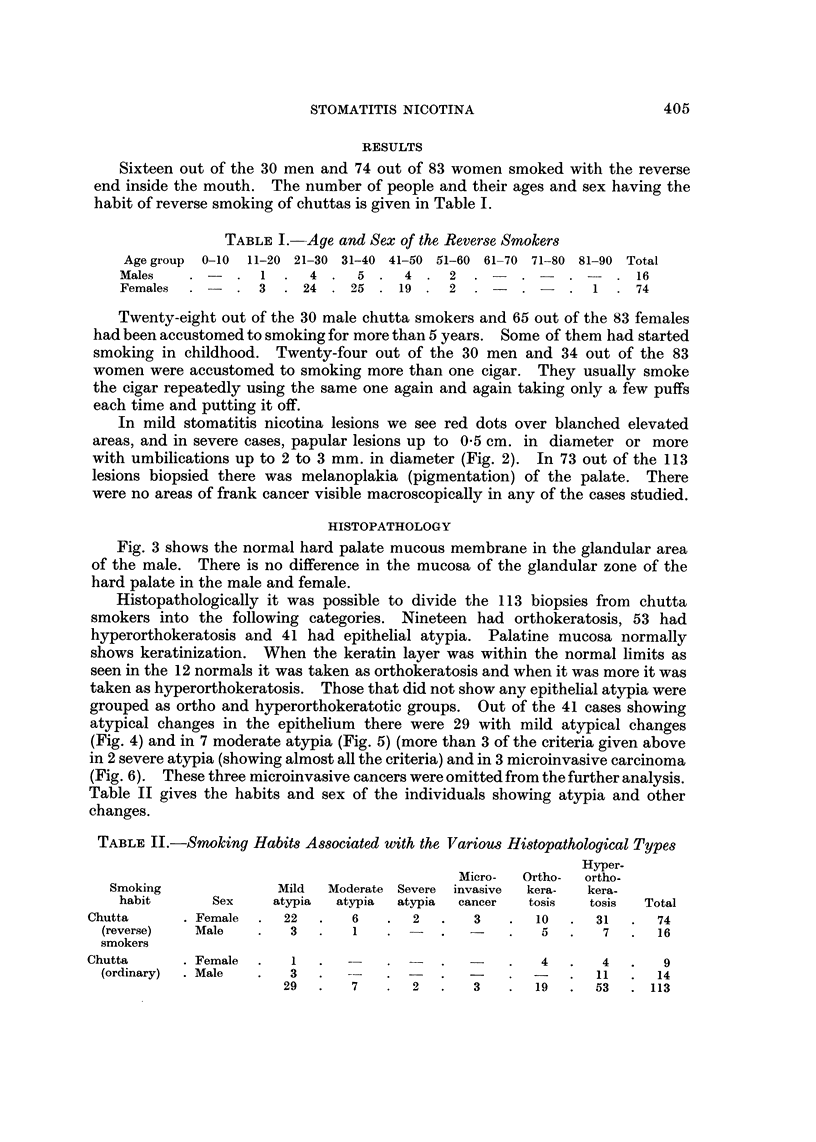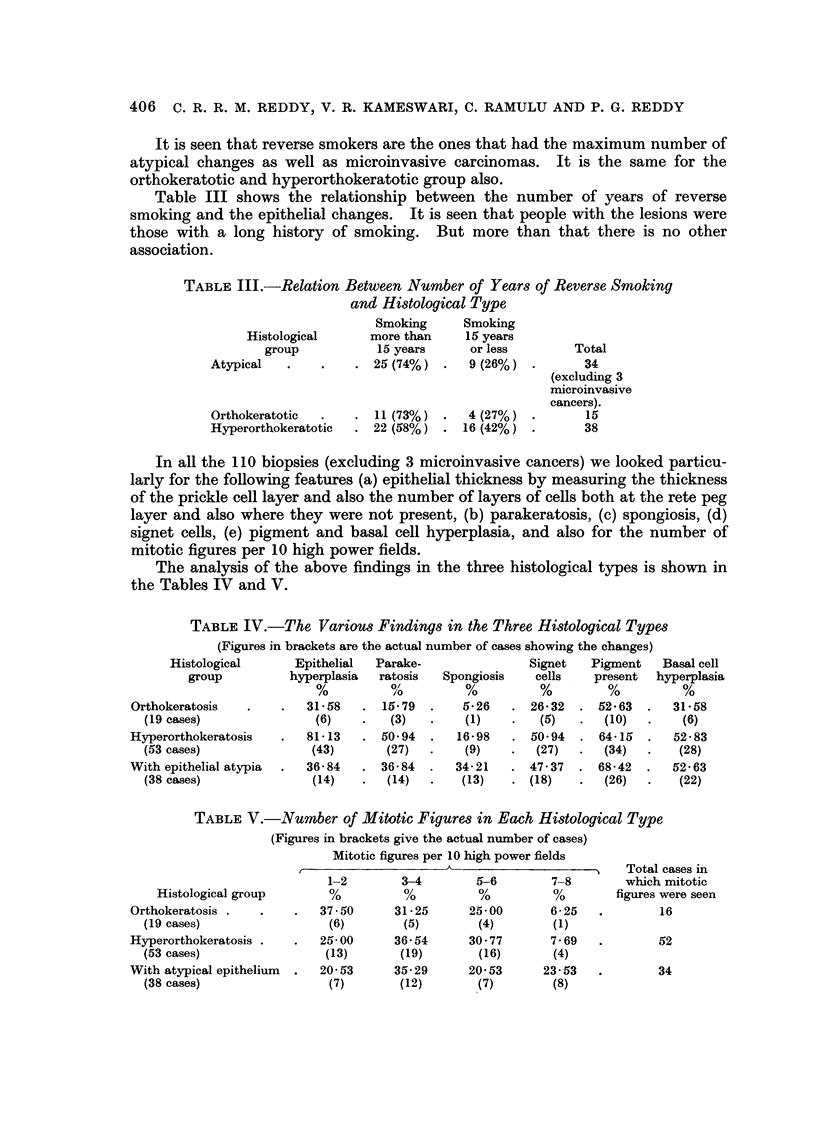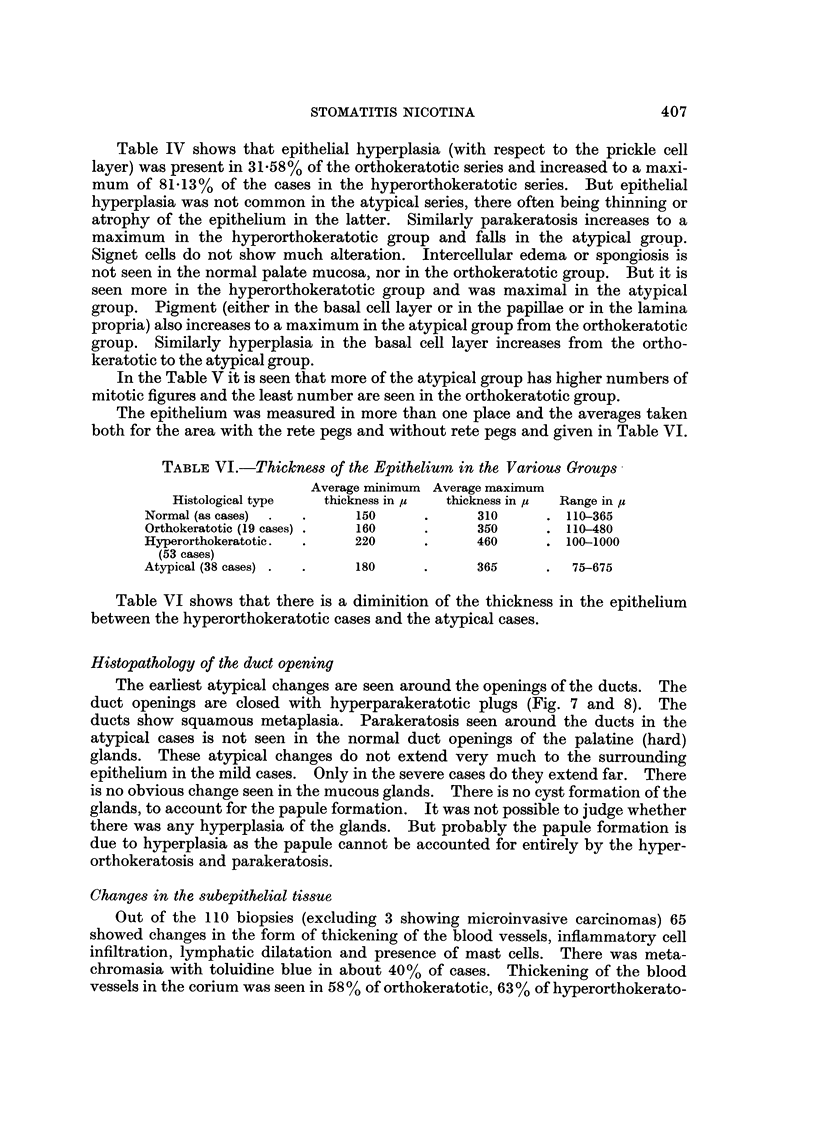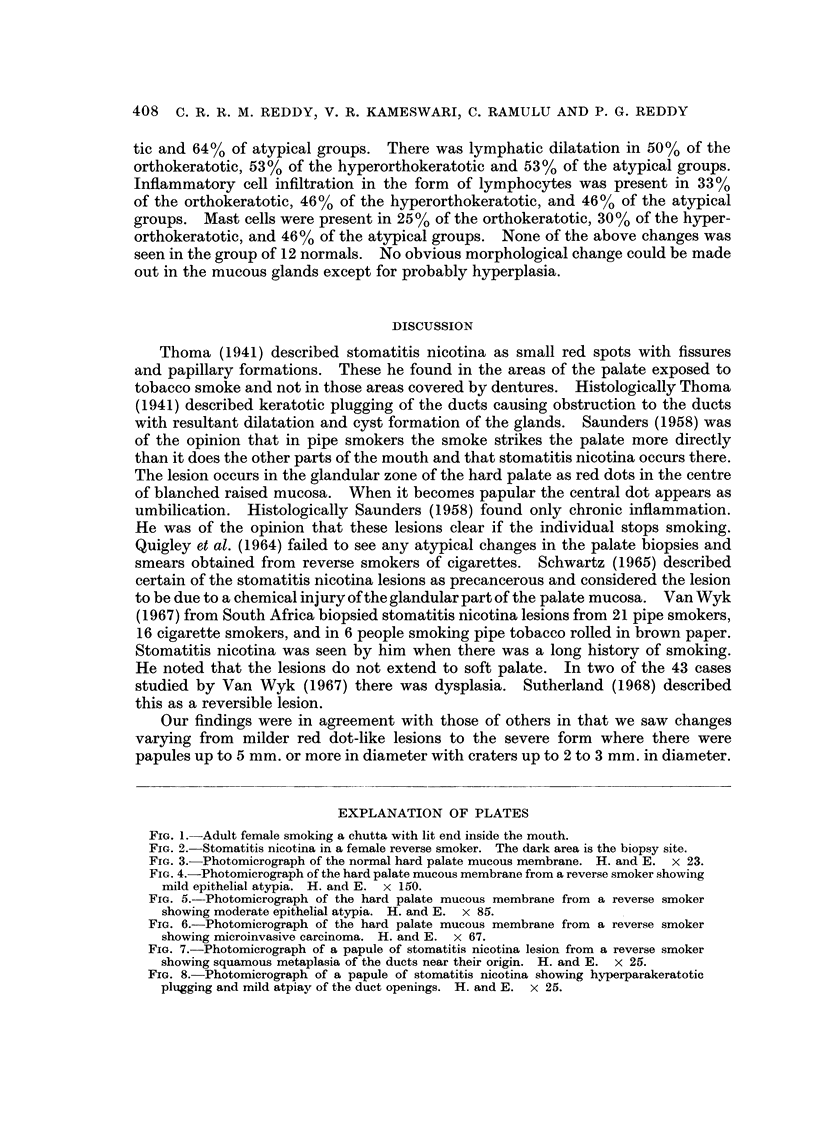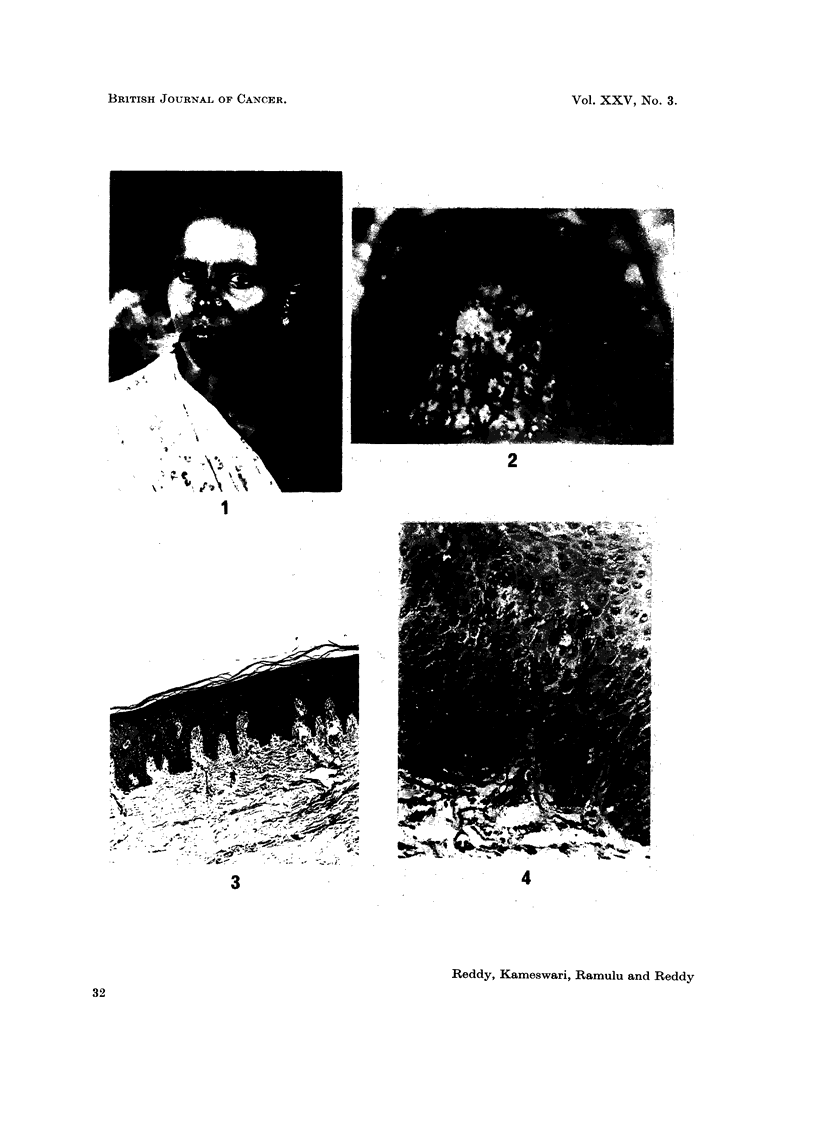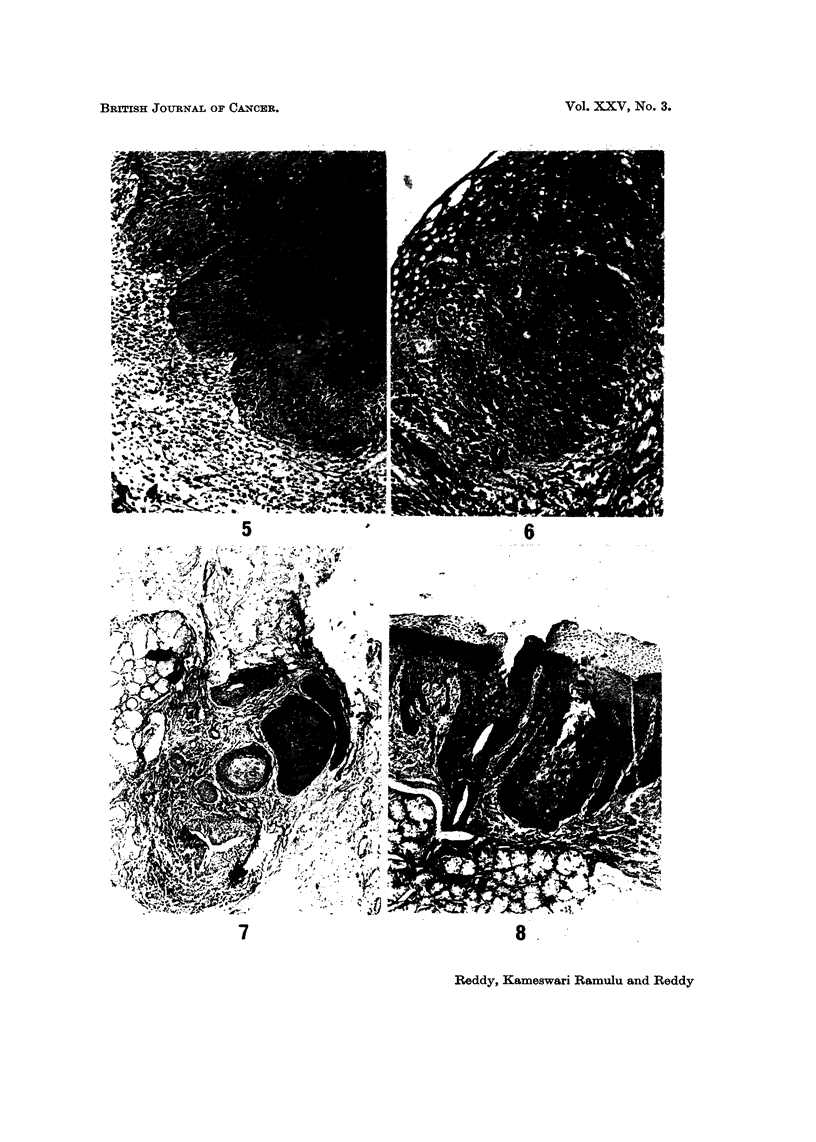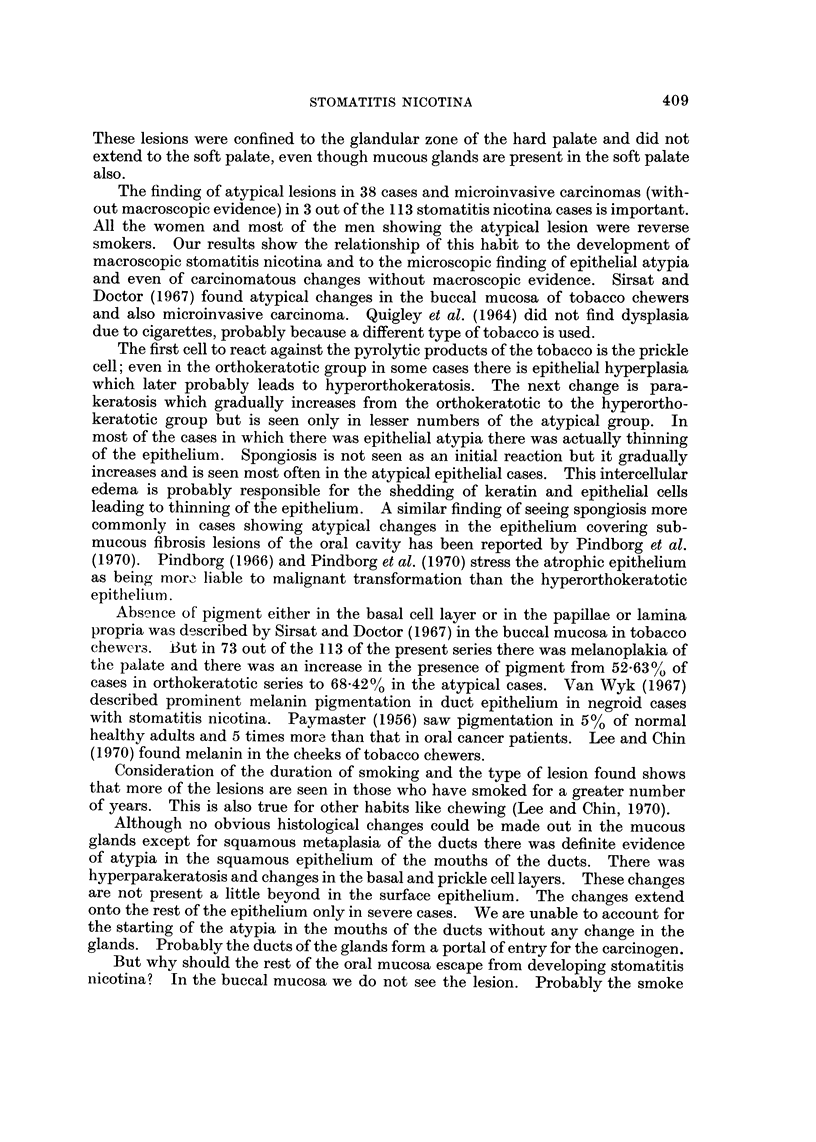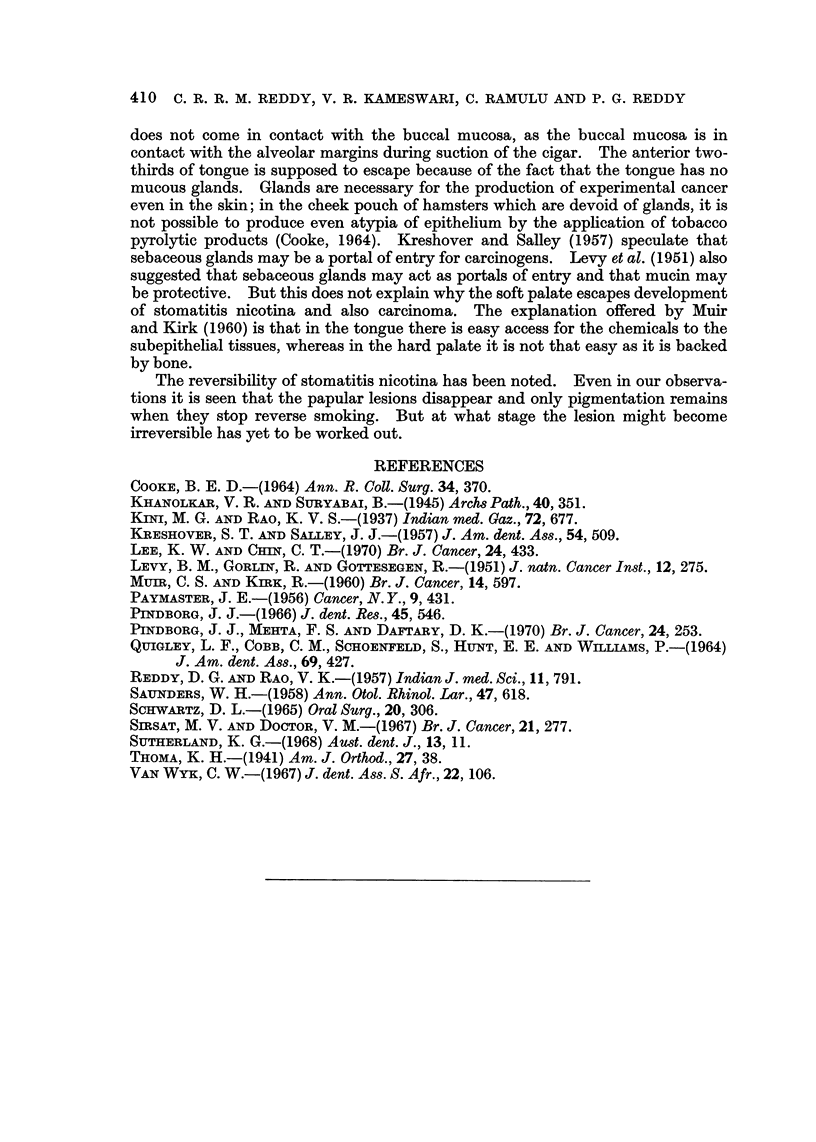# Histopathological Study of Stomatitis Nicotina

**DOI:** 10.1038/bjc.1971.51

**Published:** 1971-09

**Authors:** C. R. R. M. Reddy, V. R. Kameswari, C. Ramulu, P. G. Reddy

## Abstract

**Images:**


### HISTOPATHOLOGICAL STUDY OF STOMATITIS NICOTINA

C. R. R. M. REDDY, V. R. KAMESWARI, C. RAMULU AND P. G. REDDY

*From the Departments of Pathology and Dentistry, Andhra Medical College, Visakhapatnam, Andhra Pradesh, S. India*

Received for publication June 14, 1971

**SUMMARY.—One hundred and thirteen biopsies of the palate in people accustomed to smoking cigars, most of them with the burning end of the cigar inside the mouth, have been studied.**

**Thirty-eight of these showed mild to severe atypical changes in the epithelium. There were 19 lesions showing orthokeratosis and 53 showing hyperorthokeratosis.**

**The earliest atypical change is seen in the mouths of the ducts of the glands.**

**There were 3 cases showing microinvasive carcinomas.**

**Pigmentation is a prominent feature in these cases.**

**The papules with umbilication could be due to hyperplasia of the mucous glands.**

**It is suggested that stomatitis nicotina occurring in men and women with the habit of reverse smoking is probably precancerous because of the presence of atypical changes in the epithelium and also the finding of 3 microinvasive carcinomas without any macroscopic evidence.**

**There is no acceptable explanation why the soft palate escapes getting either stomatitis nicotina lesion or carcinoma in reverse smokers.**

THE effect of smoking on the oral cavity has been well studied for quite a number of years in the West. A peculiar method of smoking home made cigars (smoking with the burning end inside the mouth) is common in Visakhapatnam district in the east coast of India and carcinoma of the hard palate is associated with it, especially in women (Kini and Rao, 1937; Khanolkar and Suryabai, 1945; Reddy and Rao, 1957). But so far no particular effort has been made to study the changes in the palate due to this method of smoking and the histological changes produced in the palate as a result of this type of smoking.

Stomatitis nicotina, described by Thoma in 1941, is the name given to the changes in the hard palate in cigar smokers. This lesion has been studied by others such as Saunders (1958) in North America, Schwartz (1965) also in North America, Van Wyk (1967) in South Africa and Sutherland (1968) in Australia in people who smoke cigarettes and cigars. Quigley *et al.* (1964) studied the palatal changes in Caribbean and South American people some of whom smoke the cigarettes with the burning end inside the mouth. Histopathological studies have been done by some of the above workers to determine whether there are any atypical changes in the squamous epithelium of the palate as a result of stomatitis nicotina.

As the tobacco which is used by the local people to smoke in this peculiar way is different from the tobacco used for making cigarettes and cigars we felt it is worthwhile to study these lesions histopathologically to see how far they could be shown to be precancerous, if in fact they are precancerous.

### MATERIALS AND METHODS

One hundred and thirteen people belonging to the low socio-economic group with the habit of smoking home made cigars* either with or without the burning end inside the mouth (Fig. 1) who visited the Dental Out-Patient Department were studied. A biopsy was taken from the glandular portion of hard palate mucosa showing the stomatitis nicotina lesion. Mucous membrane of the hard palate was biopsied from 12 normal people who did not have any smoking, chewing or drinking habits. All the biopsies were done under local anaesthesia. Both males and females were included in the study. The clinical findings, age, sex, number of cigars etc. smoked and number of years of having the smoking habit were analysed.

The slides were stained with haematoxylin and eosin, PAS, and toluidine blue, and by Verhoeff's, Van Gieson's and Masson's trichrome methods.

### *Stomatitis nicotina*

When the glandular area of the hard palate mucosa showed papular elevations (up to 2 to 3 mm. in height) with central umbilications, with or without pigmentation of the surrounding mucosa, it was taken as stomatitis nicotina. The central umbilication could be like a red spot in the centre of a greyish or pale elevated papule about 1 to 5 mm. in diameter. Usually there were many of these papular lesions in the glandular part of hard palate mucosa. They were not present in the soft palate or in the anterior half or third of the hard palate, and they did not extend up to the alveolar margin. The following features were studied in the squamous epithelium:

*Type of keratinization:*

1. Orthokeratosis. 2. Hyperkeratosis. 3. Parakeratosis.

*Changes in the thickness of the epithelium and the cells:*
1. The thickness of the epithelium at the rete pegs and at the papillary level. 2. The number of layers of prickle cells in the rete peg area and opposite the papillary area. 3. Presence or absence of rete pegs. 4. Blunting or pointing of the rete pegs. 5. Presence or absence of a stratum granulosum and the number of cell layers in it. 6. Number of mitotic figures per 10 high power fields, 7. Presence or absence of pigment in the basal cells. 8. Presence or absence of pigment in the papillae. 9. Spongiosis or intercellular oedema. 10. Presence of signet cells.

*Epithelial atypia.* The sections were evaluated with regard to the following features. If two or more of the following were present epithelial atypia was diagnosed. 1. Irregular epithelial stratification. 2. Basal cell hyperplasia. 3. Increased mitotic figures. 4. Any abnormal mitoses. 5. Increased nuclear/cytoplasmic ratio. 6. Loss of polarity of cells. 7. Cellular and nuclear pleomorphism. 8. Hyperchromatism. 9. Keratinization of single or groups of cells in the prickle cell layer. 10. Enlarged nucleoli in epithelial cells.

* Chutta is a home made cigar and consists of bits of tobacco leaf wrapped round by a tobacco leaf. Length varies from 7·5 to 10 cm., and the diameter from 0·75 to 1 cm. The tobacco, which is sun cured and grown locally, is called " Lanka " or " Garapa ".

### RESULTS

Sixteen out of the 30 men and 74 out of 83 women smoked with the reverse end inside the mouth. The number of people and their ages and sex having the habit of reverse smoking of chuttas is given in Table I.

TABLE I.—*Age and Sex of the Reverse Smokers*

| Age group | 0–10 | 11–20 | 21–30 | 31–40 | 41–50 | 51–60 | 61–70 | 71–80 | 81–90 | Total |
|---|---|---|---|---|---|---|---|---|---|---|
| Males | — | 1 | 4 | 5 | 4 | 2 | — | — | — | 16 |
| Females | — | 3 | 24 | 25 | 19 | 2 | — | — | 1 | 74 |

Twenty-eight out of the 30 male chutta smokers and 65 out of the 83 females had been accustomed to smoking for more than 5 years. Some of them had started smoking in childhood. Twenty-four out of the 30 men and 34 out of the 83 women were accustomed to smoking more than one cigar. They usually smoke the cigar repeatedly using the same one again and again taking only a few puffs each time and putting it off.

In mild stomatitis nicotina lesions we see red dots over blanched elevated areas, and in severe cases, papular lesions up to 0·5 cm. in diameter or more with umbilications up to 2 to 3 mm. in diameter (Fig. 2). In 73 out of the 113 lesions biopsied there was melanoplakia (pigmentation) of the palate. There were no areas of frank cancer visible macroscopically in any of the cases studied.

### HISTOPATHOLOGY

Fig. 3 shows the normal hard palate mucous membrane in the glandular area of the male. There is no difference in the mucosa of the glandular zone of the hard palate in the male and female.

Histopathologically it was possible to divide the 113 biopsies from chutta smokers into the following categories. Nineteen had orthokeratosis, 53 had hyperorthokeratosis and 41 had epithelial atypia. Palatine mucosa normally shows keratinization. When the keratin layer was within the normal limits as seen in the 12 normals it was taken as orthokeratosis and when it was more it was taken as hyperorthokeratosis. Those that did not show any epithelial atypia were grouped as ortho and hyperorthokeratotic groups. Out of the 41 cases showing atypical changes in the epithelium there were 29 with mild atypical changes (Fig. 4) and in 7 moderate atypia (Fig. 5) (more than 3 of the criteria given above in 2 severe atypia (showing almost all the criteria) and in 3 microinvasive carcinoma (Fig. 6). These three microinvasive cancers were omitted from the further analysis. Table II gives the habits and sex of the individuals showing atypia and other changes.

TABLE II.—*Smoking Habits Associated with the Various Histopathological Types*

| Smoking habit | Sex | Mild atypia | Moderate atypia | Severe atypia | Micro-invasive cancer | Ortho-kera-tosis | Hyper-ortho-kera-tosis | Total |
|---|---|---|---|---|---|---|---|---|
| Chutta (reverse) smokers | Female | 22 | 6 | 2 | 3 | 10 | 31 | 74 |
| | Male | 3 | 1 | — | — | 5 | 7 | 16 |
| Chutta (ordinary) | Female | 1 | — | — | — | 4 | 4 | 9 |
| | Male | 3 | — | — | — | — | 11 | 14 |
| | | 29 | 7 | 2 | 3 | 19 | 53 | 113 |

It is seen that reverse smokers are the ones that had the maximum number of atypical changes as well as microinvasive carcinomas. It is the same for the orthokeratotic and hyperorthokeratotic group also.

Table III shows the relationship between the number of years of reverse smoking and the epithelial changes. It is seen that people with the lesions were those with a long history of smoking. But more than that there is no other association.

TABLE III.—*Relation Between Number of Years of Reverse Smoking and Histological Type*

| Histological group | Smoking more than 15 years | Smoking 15 years or less | Total |
|---|---|---|---|
| Atypical | 25 (74%) | 9 (26%) | 34 (excluding 3 microinvasive cancers). |
| Orthokeratotic | 11 (73%) | 4 (27%) | 15 |
| Hyperorthokeratotic | 22 (58%) | 16 (42%) | 38 |

In all the 110 biopsies (excluding 3 microinvasive cancers) we looked particularly for the following features (a) epithelial thickness by measuring the thickness of the prickle cell layer and also the number of layers of cells both at the rete peg layer and also where they were not present, (b) parakeratosis, (c) spongiosis, (d) signet cells, (e) pigment and basal cell hyperplasia, and also for the number of mitotic figures per 10 high power fields.

The analysis of the above findings in the three histological types is shown in the Tables IV and V.

TABLE IV.—*The Various Findings in the Three Histological Types*

(Figures in brackets are the actual number of cases showing the changes)

| Histological group | Epithelial hyperplasia % | Parakeratosis % | Spongiosis % | Signet cells % | Pigment present % | Basal cell hyperplasia % |
|---|---|---|---|---|---|---|
| Orthokeratosis | 31·58 | 15·79 | 5·26 | 26·32 | 52·63 | 31·58 |
| (19 cases) | (6) | (3) | (1) | (5) | (10) | (6) |
| Hyperorthokeratosis | 81·13 | 50·94 | 16·98 | 50·94 | 64·15 | 52·83 |
| (53 cases) | (43) | (27) | (9) | (27) | (34) | (28) |
| With epithelial atypia | 36·84 | 36·84 | 34·21 | 47·37 | 68·42 | 52·63 |
| (38 cases) | (14) | (14) | (13) | (18) | (26) | (22) |

TABLE V.—*Number of Mitotic Figures in Each Histological Type*

(Figures in brackets give the actual number of cases)

| Histological group | Mitotic figures per 10 high power fields 1–2 % | 3–4 % | 5–6 % | 7–8 % | Total cases in which mitotic figures were seen |
|---|---|---|---|---|---|
| Orthokeratosis | 37·50 | 31·25 | 25·00 | 6·25 | 16 |
| (19 cases) | (6) | (5) | (4) | (1) | |
| Hyperorthokeratosis | 25·00 | 36·54 | 30·77 | 7·69 | 52 |
| (53 cases) | (13) | (19) | (16) | (4) | |
| With atypical epithelium | 20·53 | 35·29 | 20·53 | 23·53 | 34 |
| (38 cases) | (7) | (12) | (7) | (8) | |

Table IV shows that epithelial hyperplasia (with respect to the prickle cell layer) was present in 31·58% of the orthokeratotic series and increased to a maximum of 81·13% of the cases in the hyperorthokeratotic series. But epithelial hyperplasia was not common in the atypical series, there often being thinning or atrophy of the epithelium in the latter. Similarly parakeratosis increases to a maximum in the hyperorthokeratotic group and falls in the atypical group. Signet cells do not show much alteration. Intercellular edema or spongiosis is not seen in the normal palate mucosa, nor in the orthokeratotic group. But it is seen more in the hyperorthokeratotic group and was maximal in the atypical group. Pigment (either in the basal cell layer or in the papillae or in the lamina propria) also increases to a maximum in the atypical group from the orthokeratotic group. Similarly hyperplasia in the basal cell layer increases from the orthokeratotic to the atypical group.

In the Table V it is seen that more of the atypical group has higher numbers of mitotic figures and the least number are seen in the orthokeratotic group.

The epithelium was measured in more than one place and the averages taken both for the area with the rete pegs and without rete pegs and given in Table VI.

TABLE VI.—*Thickness of the Epithelium in the Various Groups*

| Histological type | Average minimum thickness in $\mu$ | Average maximum thickness in $\mu$ | Range in $\mu$ |
|---|---|---|---|
| Normal (as cases) | 150 | 310 | 110–365 |
| Orthokeratotic (19 cases) | 160 | 350 | 110–480 |
| Hyperorthokeratotic. (53 cases) | 220 | 460 | 100–1000 |
| Atypical (38 cases) | 180 | 365 | 75–675 |

Table VI shows that there is a diminition of the thickness in the epithelium between the hyperorthokeratotic cases and the atypical cases.

*Histopathology of the duct opening*

The earliest atypical changes are seen around the openings of the ducts. The duct openings are closed with hyperparakeratotic plugs (Fig. 7 and 8). The ducts show squamous metaplasia. Parakeratosis seen around the ducts in the atypical cases is not seen in the normal duct openings of the palatine (hard) glands. These atypical changes do not extend very much to the surrounding epithelium in the mild cases. Only in the severe cases do they extend far. There is no obvious change seen in the mucous glands. There is no cyst formation of the glands, to account for the papule formation. It was not possible to judge whether there was any hyperplasia of the glands. But probably the papule formation is due to hyperplasia as the papule cannot be accounted for entirely by the hyperorthokeratosis and parakeratosis.

*Changes in the subepithelial tissue*

Out of the 110 biopsies (excluding 3 showing microinvasive carcinomas) 65 showed changes in the form of thickening of the blood vessels, inflammatory cell infiltration, lymphatic dilatation and presence of mast cells. There was metachromasia with toluidine blue in about 40% of cases. Thickening of the blood vessels in the corium was seen in 58% of orthokeratotic, 63% of hyperorthokeratotic and 64% of atypical groups. There was lymphatic dilatation in 50% of the orthokeratotic, 53% of the hyperorthokeratotic and 53% of the atypical groups. Inflammatory cell infiltration in the form of lymphocytes was present in 33% of the orthokeratotic, 46% of the hyperorthokeratotic, and 46% of the atypical groups. Mast cells were present in 25% of the orthokeratotic, 30% of the hyperorthokeratotic, and 46% of the atypical groups. None of the above changes was seen in the group of 12 normals. No obvious morphological change could be made out in the mucous glands except for probably hyperplasia.

### DISCUSSION

Thoma (1941) described stomatitis nicotina as small red spots with fissures and papillary formations. These he found in the areas of the palate exposed to tobacco smoke and not in those areas covered by dentures. Histologically Thoma (1941) described keratotic plugging of the ducts causing obstruction to the ducts with resultant dilatation and cyst formation of the glands. Saunders (1958) was of the opinion that in pipe smokers the smoke strikes the palate more directly than it does the other parts of the mouth and that stomatitis nicotina occurs there. The lesion occurs in the glandular zone of the hard palate as red dots in the centre of blanched raised mucosa. When it becomes papular the central dot appears as umbilication. Histologically Saunders (1958) found only chronic inflammation. He was of the opinion that these lesions clear if the individual stops smoking. Quigley *et al.* (1964) failed to see any atypical changes in the palate biopsies and smears obtained from reverse smokers of cigarettes. Schwartz (1965) described certain of the stomatitis nicotina lesions as precancerous and considered the lesion to be due to a chemical injury of the glandular part of the palate mucosa. Van Wyk (1967) from South Africa biopsied stomatitis nicotina lesions from 21 pipe smokers, 16 cigarette smokers, and in 6 people smoking pipe tobacco rolled in brown paper. Stomatitis nicotina was seen by him when there was a long history of smoking. He noted that the lesions do not extend to soft palate. In two of the 43 cases studied by Van Wyk (1967) there was dysplasia. Sutherland (1968) described this as a reversible lesion.

Our findings were in agreement with those of others in that we saw changes varying from milder red dot-like lesions to the severe form where there were papules up to 5 mm. or more in diameter with craters up to 2 to 3 mm. in diameter.

---

### EXPLANATION OF PLATES

FIG. 1.—Adult female smoking a chutta with lit end inside the mouth.
FIG. 2.—Stomatitis nicotina in a female reverse smoker. The dark area is the biopsy site.
FIG. 3.—Photomicrograph of the normal hard palate mucous membrane. H. and E. × 23.
FIG. 4.—Photomicrograph of the hard palate mucous membrane from a reverse smoker showing mild epithelial atypia. H. and E. × 150.
FIG. 5.—Photomicrograph of the hard palate mucous membrane from a reverse smoker showing moderate epithelial atypia. H. and E. × 85.
FIG. 6.—Photomicrograph of the hard palate mucous membrane from a reverse smoker showing microinvasive carcinoma. H. and E. × 67.
FIG. 7.—Photomicrograph of a papule of stomatitis nicotina lesion from a reverse smoker showing squamous metaplasia of the ducts near their origin. H. and E. × 25.
FIG. 8.—Photomicrograph of a papule of stomatitis nicotina showing hyperparakeratotic plugging and mild atpiay of the duct openings. H. and E. × 25.

1

2

3

4

5

6

7

8

Reddy, Kameswari Ramulu and Reddy

These lesions were confined to the glandular zone of the hard palate and did not extend to the soft palate, even though mucous glands are present in the soft palate also.

The finding of atypical lesions in 38 cases and microinvasive carcinomas (without macroscopic evidence) in 3 out of the 113 stomatitis nicotina cases is important. All the women and most of the men showing the atypical lesion were reverse smokers. Our results show the relationship of this habit to the development of macroscopic stomatitis nicotina and to the microscopic finding of epithelial atypia and even of carcinomatous changes without macroscopic evidence. Sirsat and Doctor (1967) found atypical changes in the buccal mucosa of tobacco chewers and also microinvasive carcinoma. Quigley *et al.* (1964) did not find dysplasia due to cigarettes, probably because a different type of tobacco is used.

The first cell to react against the pyrolytic products of the tobacco is the prickle cell; even in the orthokeratotic group in some cases there is epithelial hyperplasia which later probably leads to hyperorthokeratosis. The next change is parakeratosis which gradually increases from the orthokeratotic to the hyperorthokeratotic group but is seen only in lesser numbers of the atypical group. In most of the cases in which there was epithelial atypia there was actually thinning of the epithelium. Spongiosis is not seen as an initial reaction but it gradually increases and is seen most often in the atypical epithelial cases. This intercellular edema is probably responsible for the shedding of keratin and epithelial cells leading to thinning of the epithelium. A similar finding of seeing spongiosis more commonly in cases showing atypical changes in the epithelium covering submucous fibrosis lesions of the oral cavity has been reported by Pindborg *et al.* (1970). Pindborg (1966) and Pindborg *et al.* (1970) stress the atrophic epithelium as being more liable to malignant transformation than the hyperorthokeratotic epithelium.

Absence of pigment either in the basal cell layer or in the papillae or lamina propria was described by Sirsat and Doctor (1967) in the buccal mucosa in tobacco chewers. But in 73 out of the 113 of the present series there was melanoplakia of the palate and there was an increase in the presence of pigment from 52·63% of cases in orthokeratotic series to 68·42% in the atypical cases. Van Wyk (1967) described prominent melanin pigmentation in duct epithelium in negroid cases with stomatitis nicotina. Paymaster (1956) saw pigmentation in 5% of normal healthy adults and 5 times more than that in oral cancer patients. Lee and Chin (1970) found melanin in the cheeks of tobacco chewers.

Consideration of the duration of smoking and the type of lesion found shows that more of the lesions are seen in those who have smoked for a greater number of years. This is also true for other habits like chewing (Lee and Chin, 1970).

Although no obvious histological changes could be made out in the mucous glands except for squamous metaplasia of the ducts there was definite evidence of atypia in the squamous epithelium of the mouths of the ducts. There was hyperparakeratosis and changes in the basal and prickle cell layers. These changes are not present a little beyond in the surface epithelium. The changes extend onto the rest of the epithelium only in severe cases. We are unable to account for the starting of the atypia in the mouths of the ducts without any change in the glands. Probably the ducts of the glands form a portal of entry for the carcinogen.

But why should the rest of the oral mucosa escape from developing stomatitis nicotina? In the buccal mucosa we do not see the lesion. Probably the smoke does not come in contact with the buccal mucosa, as the buccal mucosa is in contact with the alveolar margins during suction of the cigar. The anterior two-thirds of tongue is supposed to escape because of the fact that the tongue has no mucous glands. Glands are necessary for the production of experimental cancer even in the skin; in the cheek pouch of hamsters which are devoid of glands, it is not possible to produce even atypia of epithelium by the application of tobacco pyrolytic products (Cooke, 1964). Kreshover and Salley (1957) speculate that sebaceous glands may be a portal of entry for carcinogens. Levy *et al.* (1951) also suggested that sebaceous glands may act as portals of entry and that mucin may be protective. But this does not explain why the soft palate escapes development of stomatitis nicotina and also carcinoma. The explanation offered by Muir and Kirk (1960) is that in the tongue there is easy access for the chemicals to the subepithelial tissues, whereas in the hard palate it is not that easy as it is backed by bone.

The reversibility of stomatitis nicotina has been noted. Even in our observations it is seen that the papular lesions disappear and only pigmentation remains when they stop reverse smoking. But at what stage the lesion might become irreversible has yet to be worked out.

### REFERENCES

Cooke, B. E. D.—(1964) *Ann. R. Coll. Surg.* **34**, 370.
Khanolkar, V. R. and Suryabai, B.—(1945) *Archs Path.*, **40**, 351.
Kini, M. G. and Rao, K. V. S.—(1937) *Indian med. Gaz.*, **72**, 677.
Kreshover, S. T. and Salley, J. J.—(1957) *J. Am. dent. Ass.*, **54**, 509.
Lee, K. W. and Chin, C. T.—(1970) *Br. J. Cancer*, **24**, 433.
Levy, B. M., Gorlin, R. and Gottesegen, R.—(1951) *J. natn. Cancer Inst.*, **12**, 275.
Muir, C. S. and Kirk, R.—(1960) *Br. J. Cancer*, **14**, 597.
Paymaster, J. E.—(1956) *Cancer, N.Y.*, **9**, 431.
Pindborg, J. J.—(1966) *J. dent. Res.*, **45**, 546.
Pindborg, J. J., Mehta, F. S. and Daftary, D. K.—(1970) *Br. J. Cancer*, **24**, 253.
Quigley, L. F., Cobb, C. M., Schoenfeld, S., Hunt, E. E. and Williams, P.—(1964) *J. Am. dent. Ass.*, **69**, 427.
Reddy, D. G. and Rao, V. K.—(1957) *Indian J. med. Sci.*, **11**, 791.
Saunders, W. H.—(1958) *Ann. Otol. Rhinol. Lar.*, **47**, 618.
Schwartz, D. L.—(1965) *Oral Surg.*, **20**, 306.
Sirsat, M. V. and Doctor, V. M.—(1967) *Br. J. Cancer*, **21**, 277.
Sutherland, K. G.—(1968) *Aust. dent. J.*, **13**, 11.
Thoma, K. H.—(1941) *Am. J. Orthod.*, **27**, 38.
Van Wyk, C. W.—(1967) *J. dent. Ass. S. Afr.*, **22**, 106.